# Experimental and Theoretical Studies on the Kinetics and Mechanism of the C_3_H_8_/C_3_D_8_ + Cl Reaction

**DOI:** 10.3390/molecules30224406

**Published:** 2025-11-14

**Authors:** Łukasz Fojcik, Grzegorz Mierzwa, Zdzisław Latajka, Dariusz Stanisław Sarzyński

**Affiliations:** 1 Institute of Environmental Protection—National Research Institute, ul. Słowicza 32, 02-170 Warszawa, Poland; lukasz.fojcik@ios.edu.pl (Ł.F.); grzegorz.mierzwa@ios.edu.pl (G.M.); 2 Faculty of Chemistry, University of Wrocław, ul. Joliot-Curie 14, 50-383 Wrocław, Poland; zdzislaw.latajka@uwr.edu.pl; 3 Department and Institute of Basic Chemical Sciences, Wroclaw Medical University, ul. Borowska 211a, 50-556 Wrocław, Poland

**Keywords:** Cl atom reactions, hydrogen/deuterium abstraction, KIE, propane, propane-d8, pre-reactive complex, transition state (TS), intrinsic reaction coordinate (IRC), *L-parameter*, barrierless entrance channel, MP2/aug-cc-pVDZ, Gaussian 16 (G16) computations

## Abstract

An experimental and theoretical investigation of the reaction between chlorine atoms and propane/deuterated propane (C_3_H_8_/C_3_D_8_) was performed. The experimental work aimed to determine absolute and site-specific rate constants for hydrogen and deuterium abstraction in the Cl + C_3_H_8_/C_3_D_8_ system. Measurements were conducted using the relative rate method at three temperatures between 298 and 387 K. Total rate constants for H/D abstraction by chlorine, as well as individual rate constants for abstraction from primary and secondary carbon sites, were obtained. The kinetic data for H abstraction agree well with previously reported literature values, confirming the reliability of the experimental approach. Notably, rate constants for the C_3_D_8_ + Cl reaction were determined for the first time, and the consistency of these results supports the reliability of the newly derived kinetic parameters. In the theoretical part of the study, hydrogen/deuterium abstraction from propane by atomic chlorine was analyzed within an atmospheric-chemistry context to clarify temperature dependence and site selectivity. Stationary points (SC, TS, PC, reactants, products) were optimized at MP2/aug-cc-pVDZ and verified by harmonic frequencies and intrinsic reaction-coordinate analyses. Eyring transition-state theory yielded 298–550 K rate constants with activation free energies referenced to SC. Our calculations indicate entrance-channel complex formation and effectively barrierless progress for most pathways; a small barrier appears only for RD1′. *L-parameter* evaluation classifies TS2 as reactant-like, and branching ratios identify –CH_2_– abstraction (RX2) as dominant. These findings align with the experimental data.

## 1. Introduction

Non-methane hydrocarbons (NMHCs) are key contributors to the formation of secondary organic aerosols and atmospheric ozone, significantly affecting air quality and climate change [1]. Among these, ethane and propane are the most abundant in the atmosphere [2]. Although their concentrations declined annually in the Northern Hemisphere between 2009 and 2015, they remain the dominant NMHCs, strongly influencing tropospheric ozone levels—a major air pollutant and greenhouse gas [2].

Atomic chlorine is a critical oxidant in the troposphere. Large quantities originate from sea salt aerosols (SSA), halogen-containing gases released into the troposphere, and stratospheric leakage. It also cycles through various inorganic non-radical reservoirs [3,4,5,6,7]. Additional sources include biomass burning, coal combustion, waste incineration, industrial emissions, road salt application, and organochlorine compound release from oceans [8,9,10,11,12]. In the troposphere, atomic chlorine reacts with methane and other volatile organic compounds (VOCs) [13], as well as with dimethyl sulfide (DMS) [14,15] and mercury [16].

Deuterated compounds, such as fully or selectively deuterated propane, provide valuable insights into kinetic isotope effects by influencing reaction rates in atmospheric chemistry. The greater mass of deuterium slows reactions with key atmospheric oxidants, including OH and Cl. This effect is particularly useful for modeling changes in tropospheric composition and evaluating the role of pollutants in processes like ozone formation and VOC degradation [17].

Despite the limited data on the reaction of deuterated propane with atomic chlorine, any available information on its kinetics and kinetic isotope effect (KIE) would be crucial for refining atmospheric models.

Although the kinetics of reactions involving alkanes and atomic chlorine have been widely studied, investigations focusing on deuterated analogs remain relatively scarce. In a notable contribution to this field, Hitsuda et al. examined the reaction between fully deuterated propane (C_3_D_8_) and atomic chlorine at 298 K [18]. Utilizing laser photolysis in combination with vacuum ultraviolet laser-induced fluorescence spectroscopy, they determined the rate constants for the reactions of electronically excited chlorine atoms, Cl*(^2^P_1_/_2_), and ground-state chlorine atoms, Cl(^2^P_3_/_2_), with C_3_D_8_. The measured rate constants at 298 K were (0.0 ± 1.5) × 10^−10^ cm^3^·molecule^−1^·s^−1^ for Cl*(^2^P_1_/_2_) and (0.86 ± 0.05) × 10^−10^ cm^3^·molecule^−1^·s^−1^ for Cl(^2^P_3_/_2_). A comparison of the rate constants for the Cl + C_3_H_8_ and Cl + C_3_D_8_ reaction systems yielded a kinetic isotope effect (KIE) of 1.55 ± 0.12 [18].

Given the limited availability of kinetic data for the reaction between deuterated propane and atomic chlorine, we investigated the kinetics of these processes using both experimental and theoretical approaches. The experimental work focused on determining the rate constants for deuterium atom abstraction from individual carbon sites within the propane molecule, as well as evaluating the associated kinetic isotope effects (KIEs).

The primary objective of the theoretical studies was to elucidate the underlying reaction mechanisms and kinetics. These studies provide insights into reaction pathways, energy barriers, and the selectivity of chlorine atoms toward different hydrogen positions within the propane molecule. Such knowledge is crucial for accurately modeling atmospheric chemical processes and for developing more efficient and environmentally sustainable industrial applications.

## 2. Results and Discussion

In the experimental studies, the relative rate method was utilized to determine the rate constants for the abstraction of H/D-atoms from the C_3_H_8_/C_3_D_8_ molecule by atomic chlorine.

The overall rate constant of the reaction was determined using the relative rate method based on the monitoring of substrate decay. In contrast, the relative rate method based on the detection of specific reaction products was employed to determine the rate constants for hydrogen or deuterium atom abstraction from individual carbon atoms in the propane molecule. The following text provides a concise description of both measurement methods.

In the relative rate method based on substrate decay, the reaction rate constant is determined relative to a reference reaction, for which the rate constant value is known and well-documented. The following derivation of the formula used in the relative rate method based on substrate decay is demonstrated using the reaction of C_3_H_8_ + Cl (the studied reaction) and C_2_H_6_ + Cl (the reference reaction) as an example. The studied and reference reactions can be represented as follows:

(1) $$C_{3}H_{8}+Cl\overset{k_{H}}{\to }C_{3}H_{7}+HCl\;\mathrm{Studied}\;\mathrm{reaction}$$

(2) $$C_{2}H_{6}+Cl\overset{k_{2}}{\to }C_{2}H_{5}+HCl\;\mathrm{Reference}\;\mathrm{reaction}$$

For fully deuterated propane, an analogous expression can be written as follows:

$$C_{3}D_{8}+Cl\overset{k_{D}}{\to }C_{3}D_{7}+DCl\;\mathrm{Studied}\;\mathrm{reaction}$$

From the above scheme of the reactions, it follows that both reactions must share a common substrate (Cl), and the reverse reactions are negligible.

The rate equations for reactions (1) and (2) are expressed as follows:

(3) $$\frac{d\left[C_{3}H_{8}\right]}{dt}=-k_{H}\cdot \left[C_{3}H_{8}\right]\cdot \left[Cl\right]$$

(4) $$\frac{d\left[C_{2}H_{6}\right]}{dt}=-k_{2}\cdot \left[C_{2}H_{6}\right]\cdot \left[Cl\right]$$

Dividing Equation (3) by Equation (4) side by side and separating the variables, we arrive at the following equation:

(5) $$\frac{d\left[C_{3}H_{8}\right]}{\left[C_{3}H_{8}\right]}=\frac{k_{H}}{k_{2}}\cdot \frac{d\left[C_{2}H_{6}\right]}{\left[C_{2}H_{6}\right]}$$

Integrating Equation (5) from the initial concentrations of the substrates [C_3_H_8_]_0_ and [C_2_H_6_]_0_ to the concentrations at time *t*, [C_3_H_8_]_t_ and [C_2_H_6_]_t_, we obtain:

(6) $$ln\frac{{\left[C_{3}H_{8}\right]}_{0}}{{\left[C_{3}H_{8}\right]}_{t}}=\frac{k_{H}}{k_{2}}\cdot ln\frac{{\left[C_{2}H_{6}\right]}_{0}}{{\left\lceil C_{2}H_{6}\right\rceil }_{t}}$$

From Equation (6), it follows that the relationship between ln([C_3_H_8_]_0_/[C_3_H_8_]_t_) and ln([C_2_H_6_]_0_/[C_2_H_6_]_t_) is linear, with a slope of k_H_/k_2_. Repeating the experiment with varying degrees of conversion for C_3_H_8_ and C_2_H_6_ allows for determining a linear relationship between ln([C_3_H_8_]_0_/[C_3_H_8_]_t_) and ln([C_2_H6]_0_/[C_2_H_6_]_t_), from which the slope k_H_/k_2_ can be derived. Multiplying this slope by the absolute rate constant of C_2_H_6_ + Cl yields the absolute value of k_H_.

In the case of deuterated propane, Equation (6) takes on an analogous form:

(7) $$ln\frac{{\left[C_{3}D_{8}\right]}_{0}}{{\left[C_{3}D_{8}\right]}_{t}}=\frac{k_{D}}{k_{2}}\cdot ln\frac{{\left[C_{2}H_{6}\right]}_{0}}{{\left\lceil C_{2}H_{6}\right\rceil }_{t}}$$

During the determination of the overall rate constant for the reaction C_3_D_8_ + Cl, the reaction C_2_H_6_ + Cl was also utilized as a reference reaction.

The relative rate method, based on monitoring the formation of reaction products, was employed to determine the rate constants for the abstraction of H or D atoms from specific carbon atoms in propane. The following derivation of the formula is conducted for the abstraction of a H atom from a primary carbon in the propane molecule. The reference reaction is C_2_H_6_ with Cl. After the abstraction of a H atom, the resulting radicals react immediately with Cl_2_, which is present in large excess in the reaction system. This is described by the reactions below:

(8) $${CH}_{3}{CH}_{2}{CH}_{3}+Cl\overset{k_{H,1}}{\to }{CH}_{3}{CH}_{2}{CH}_{2}+HCl$$

(9) $${CH}_{3}{CH}_{2}CH_{2}+{Cl}_{2}\overset{k_{9}}{\to }{CH}_{3}{CH}_{2}{CH}_{2}Cl+Cl$$

(2) $$C_{2}H_{6}+Cl\overset{k_{2}}{\to }C_{2}H_{5}+HCl\;\;\;\;\;$$

(10) $${CH}_{3}{CH}_{2}+{Cl}_{2}\overset{k_{10}}{\to }{CH}_{3}{CH}_{2}Cl+Cl$$

Based on the reaction scheme above, the following equations for the reaction rates of 1-chloropropane and chloroethane formation can be written:

(11) $$\frac{d\left[{CH}_{3}{CH}_{2}{CH}_{2}Cl\right]}{dt}=k_{9}\cdot \left[{CH}_{3}{CH}_{2}{CH}_{2}\right]\cdot \left[{Cl}_{2}\right]$$

(12) $$\frac{d\left[{CH}_{3}{CH}_{2}Cl\right]}{dt}=k_{10}\cdot \left[{CH}_{3}{CH}_{2}\right]\cdot \left[{Cl}_{2}\right]\;\;\;\;$$

Both equations above include the concentrations of propyl and ethyl radicals. These concentrations can be eliminated by using the rate equations for these radicals and considering that their concentrations reach a steady state, i.e., they remain constant over time:

(13) $$\frac{d\left[{CH}_{3}{CH}_{2}{CH}_{2}\right]}{dt}=0=k_{H,1}\cdot \left[{CH}_{3}{CH}_{2}{CH}_{3}\right]\cdot \left[Cl\right]-k_{9}\cdot \left[{CH}_{3}{CH}_{2}{CH}_{2}\right]\cdot \left[{Cl}_{2}\right]$$

(14) $$\frac{d\left[{CH}_{3}{CH}_{2}\right]}{dt}=0=k_{2}\cdot \left[C_{2}H_{6}\right]\cdot \left[Cl\right]-k_{10}\cdot \left[{CH}_{3}{CH}_{2}\right]\cdot \left[{Cl}_{2}\right]$$

By calculating the concentrations of propyl radicals from Equation (13) and ethyl radicals from Equation (14), and substituting them into Equations (11) and (12), we obtain:

(15) $$\frac{d\left[{CH}_{3}{CH}_{2}{CH}_{2}Cl\right]}{dt}=k_{H,1}\cdot \left[C_{3}H_{8}\right]\cdot \left[Cl\right]$$

(16) $$\frac{d\left[{CH}_{3}{CH}_{2}Cl\right]}{dt}=k_{2}\cdot \left[C_{2}H_{6}\right]\cdot \left[Cl\right]$$

Dividing Equation (15) by Equation (16) on both sides and separating the variables, we obtain:

(17) $$d\left[{CH}_{3}{CH}_{2}{CH}_{2}Cl\right]=\frac{k_{H,1}}{k_{2}}\cdot \frac{\left[C_{3}H_{8}\right]}{\left[C_{2}H_{6}\right]}\cdot d\left[{CH}_{3}{CH}_{2}Cl\right]$$

In the method of relative reaction rates based on monitoring product formation, the degree of substrate conversion must remain very low to prevent the formation of secondary reaction products. Under such experimental conditions, we can assume that [C_3_H_8_]/[C_2_H_6_] is, to a good approximation, equal to [C_3_H_8_]_0_/[C_2_H_6_]_0_.

In that case, after integration, Equation (17) takes the form:

(18) $${\left[{CH}_{3}{CH}_{2}{CH}_{2}Cl\right]}_{t}=\frac{k_{H,1}}{k_{2}}\cdot \frac{{\left[C_{3}H_{8}\right]}_{0}}{{\left[C_{2}H_{6}\right]}_{0}}\cdot {\left[C_{2}H_{5}Cl\right]}_{t}$$

By transforming Equation (18), a final formula is obtained, allowing the calculation of the ratio of reaction rate constants based on the determination of the final concentrations of reaction products:

(19) $$\frac{k_{H,1}}{k_{2}}=\frac{{\left[{CH}_{3}{CH}_{2}{CH}_{2}Cl\right]}_{t}}{{\left[C_{2}H_{5}Cl\right]}_{t}}\cdot \frac{{\left[C_{2}H_{6}\right]}_{0}}{{\left[C_{3}H_{8}\right]}_{0}}$$

The reaction of hydrogen abstraction from the secondary carbon in the propane molecule is represented by Equation (20), while the abstraction of deuterium from the primary and secondary carbons in the fully deuterated propane molecule is described by Equations (21) and (22), respectively:

(20) $${CH}_{3}{CH}_{2}{CH}_{3}+Cl\overset{k_{H,2}}{\to }{CH}_{3}CH{CH}_{3}+HCl$$

(21) $${CD}_{3}{CD}_{2}{CD}_{3}+Cl\overset{k_{D,1}}{\to }{CD}_{3}{CD}_{2}{CD}_{2}+DCl$$

(22) $${CD}_{3}{CD}_{2}{CD}_{3}+Cl\overset{k_{D,2}}{\to }{CD}_{3}CD{CD}_{3}+DCl$$

The final formulas derived for the above reactions are represented by Equations (23)–(25), respectively:

(23) $$\frac{k_{H,2}}{k_{2}}=\frac{{\left[{CH}_{3}CHCl{CH}_{3}\right]}_{t}}{{\left[C_{2}H_{5}Cl\right]}_{t}}\cdot \frac{{\left[C_{2}H_{6}\right]}_{0}}{{\left[C_{3}H_{8}\right]}_{0}}$$

(24) $$\frac{k_{D,1}}{k_{2}}=\frac{{\left[{CD}_{3}CD_{2}{CD}_{2}Cl\right]}_{t}}{{\left[C_{2}H_{5}Cl\right]}_{t}}\cdot \frac{{\left[C_{2}H_{6}\right]}_{0}}{{\left[C_{3}D_{8}\right]}_{0}}$$

(25) $$\frac{k_{D,2}}{k_{2}}=\frac{{\left[{CD}_{3}CDCl{CD}_{3}\right]}_{t}}{{\left[C_{2}H_{5}Cl\right]}_{t}}\cdot \frac{{\left[C_{2}H_{6}\right]}_{0}}{{\left[C_{3}D_{8}\right]}_{0}}$$

The reference reaction (2) was also employed in the determination of rate constants for deuterium atom abstraction.

It must be ensured that, in the relative rate method based on the determination of concentrations of respective reaction products, the experiment is conducted to avoid the formation of secondary reaction products. If such products are formed, the corresponding experimental data must be disregarded.

All experiments were performed in the temperature range of 298 to 387 K and at a total pressure of 100 Torr, using the C_2_H_6_ + Cl reaction as the reference. There is a wealth of experimental data, computational studies, and literature reviews available on the C_2_H_6_ + Cl reaction. In our study, we utilized the latest experimental data combined with a focused literature review by Hickson et al. [19]. In the temperature range of 48 to 800 K, the authors determined the following temperature dependence for the rate constant of hydrogen atom abstraction from ethane by a chlorine atom:

(26) $$k_{C_{2}H_{6}+Cl}\left(T\right)=\left(5.91\pm 1.26\right)\cdot 10^{-11}\cdot {\left(\frac{T}{298\;K}\right)}^{\left(0.27\pm 0.03\right)}\cdot e^{\frac{\left[\left(-0.12\pm 0.07\right)kJ/mol\right]}{RT}}\;{cm}^{3}{\mathrm{molecule}}^{-1}s^{-1}$$

**Figure 1:**
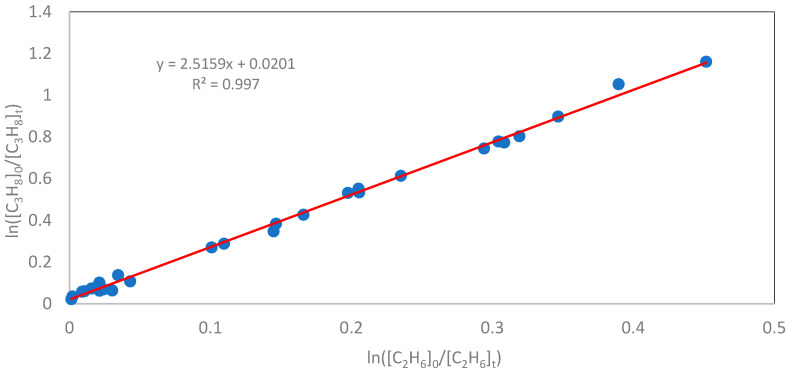
The obtained linear relationship for ln([C_3_H_8_]_0_/[C_3_H_8_]_t_) as a function of ln([C_2_H_6_]_0_/[C_2_H_6_]_t_) was determined at a temperature of 298 K and a total pressure of 100 Torr. The slope of the line, according to Equation (6), is equal to k_H_/k_2_.

The experimentally determined total rate constants and the rate constants for the abstraction of a hydrogen atom from the primary and secondary carbon atoms in the propane molecule by a chlorine atom at 298, 334, and 387 K are presented in Table 1. Figure 1 shows the plot of relationship (6) obtained at different degrees of substrate conversion at a temperature of 298 K and a total pressure of 100 Torr. The slope coefficients obtained for the hydrogen atom abstraction reaction, as described by Equation (6), together with the calculated total reaction rate constants at 298, 334, and 387 K, are summarized in Table 1 (columns 2 and 3).

**Figure 2:**
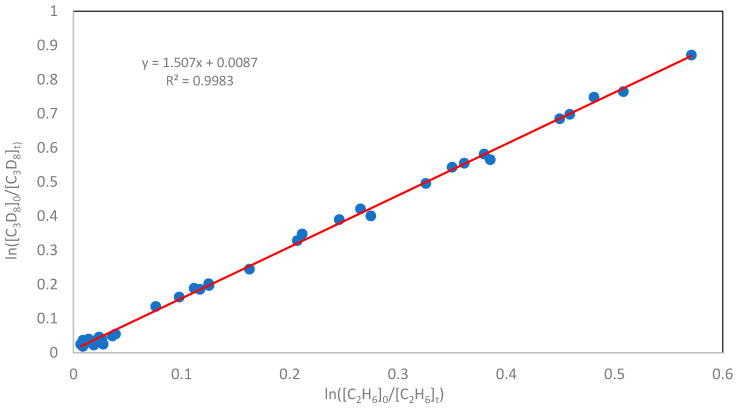
The linear relationship between ln([C_3_D_8_]_0_/[C_3_D_8_]ₜ) and ln([C_2_H_6_]_0_/[C_2_H_6_]ₜ), determined at 298 K and a total pressure of 100 Torr. The slope of the line, according to Equation (7), is equal to k_D_/k_2_.

Kinetic parameters for the deuterium abstraction from fully deuterated propane (C_3_D_8_) were determined under the same experimental conditions and at the same temperatures as those used for the hydrogen abstraction from propane (C_3_H_8_); the results are summarized in Table 2. As shown in Figure 2, a linear correlation was observed between ln([C_3_D_8_]_0_/[C_3_D_8_]ₜ) and ln([C_2_H_6_]_0_/[C_2_H_6_]ₜ), obtained over a range of substrate conversions at 298 K and a total pressure of 100 Torr.

The relative rates of H/D atom abstraction determined at 298, 334, and 387 K for C_3_H_8_ and C_3_D_8_ enabled a reliable evaluation of the kinetic isotope effect (KIE) for the investigated reaction. The calculated KIE values are summarized in Table 3.

It is instructive to compare the total rate constants for hydrogen/deuterium atom abstraction, determined using Equations (6) and (7), with those calculated as the sum of the individual rate constants for abstraction from primary and secondary carbon atoms in propane (i.e., k_H,1_ + k_H,2_ and k_D,1_ + k_D,2_, respectively).

A comparison of the overall rate constants (column 3 in Table 1) with the summed values for hydrogen abstraction from primary and secondary sites (column 8 in Table 1) shows differences of about 4%, 0%, and 3% at 298, 334, and 387 K, respectively. For deuterium abstraction (columns 3 and 8 in Table 2), the corresponding differences are approximately 5%, 5%, and 3% at the same temperatures.

These results indicate that only hydrogen or deuterium atom abstraction occurs within the reaction system. Notably, the total rate constants obtained as the sum of the individual abstraction steps are consistently lower than those derived from Equations (6) and (7), likely due to experimental factors associated with Equations (19)–(22).

In the relative rate method, which relies on quantifying primary reaction products, the formation of secondary products should ideally be avoided. However, this is not always achievable. The presence of secondary products below the detection limit of the employed thermal conductivity detector (TCD) cannot be entirely excluded. Such undetected products may slightly alter the measured concentrations of the primary products, introducing small deviations in the rate constant ratios derived.

Overall, these observations demonstrate that, for determining total reaction rate constants, the relative rate method based on monitoring substrate concentrations (Equations (6) and (7)) provides more reliable and internally consistent results.

**Figure 3:**
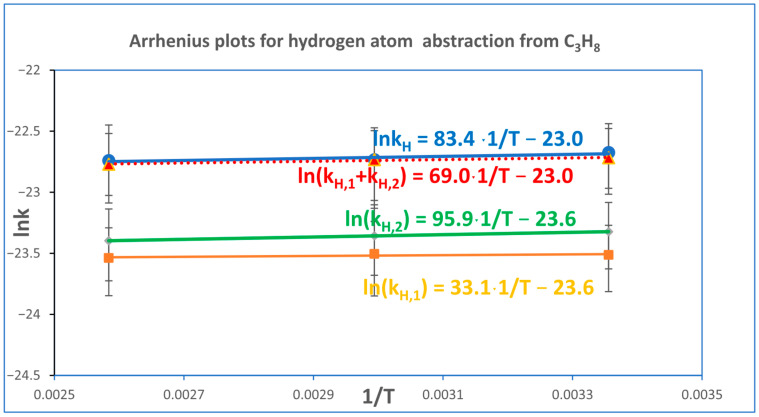
Arrhenius plots for the hydrogen abstraction reaction from propane by a chlorine atom. The top panel shows the overall reaction rate constant obtained from Equation (6). The second panel displays the summed rate constants for abstraction from primary and secondary carbon atoms, while the third and bottom panels correspond to abstraction from secondary and primary carbon atoms, respectively. Error bars represent the combined uncertainty in determining the rate constants and the propagated error associated with the reference rate constants, as calculated using Equation (26).

Figure 3 presents the Arrhenius plots for the hydrogen atom abstraction reaction from propane by a chlorine atom. The top panel shows the Arrhenius dependence of the overall rate constant determined using Equation (6). The second panel depicts the Arrhenius plot obtained by summing the rate constants for hydrogen abstraction from both primary and secondary carbon atoms. The third and fourth panels correspond to abstraction from secondary and primary carbon atoms, respectively.

**Figure 4:**
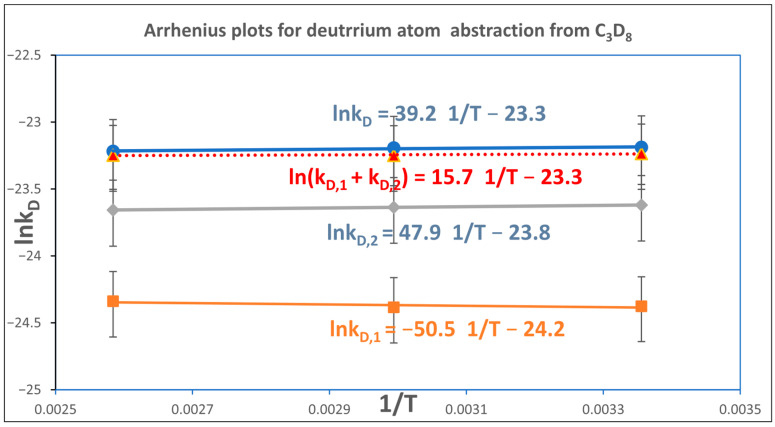
Arrhenius plots for deuterium abstraction from fully deuterated propane (C_3_D_8_) by a chlorine atom. The top panel shows the overall reaction rate constant obtained from Equation (7). The second panel displays the summed rate constants for abstraction from primary and secondary carbon atoms, while the third and bottom panels correspond to abstraction from secondary and primary carbon atoms, respectively. Error bars represent the combined uncertainty in determining the rate constants and the propagated error associated with the reference rate constants, as calculated using Equation (26).

The corresponding Arrhenius relationships for deuterium abstraction from fully deuterated propane (C_3_D_8_) are presented in Figure 4.

The kinetics of hydrogen atom abstraction from the primary and secondary carbon sites in propane by chlorine atoms were previously investigated by one of the present authors [20]. Here, we compare our relative rate constants with those earlier results. Both studies employed the C_2_H_6_ + Cl reaction as a reference and were performed at 298 K using the same apparatus. The previously reported relative rate constants—1.06 ± 0.03 for the primary site and 1.31 ± 0.03 for the secondary—are in excellent agreement with the present values of 1.09 ± 0.03 and 1.32 ± 0.04, differing by less than 3% and 1%, respectively. Measurements of the C_3_D_8_ + Cl reaction under identical conditions further confirm the consistency and reproducibility of our approach.

Earlier experimental data for the C_3_H_8_ + Cl reaction were consistent with measurements reported up to 2002; therefore, our discussion focuses on studies published thereafter. In 2006, Choi and co-workers employed laser photolysis combined with infrared detection of HCl to determine a total rate constant of (1.22 ± 0.03) × 10^−10^ cm^3^ molecule^−1^ s^−1^ at 298 K [21]. This value is approximately 16% lower than that obtained from our substrate-decay experiment and about 11% lower than the sum of the site-specific rate constants for hydrogen abstraction from the primary and secondary carbon atoms (Table 1). In contrast, the branching ratio for abstraction from the primary carbon site shows excellent agreement: Choi et al. reported (48 ± 3)%, whereas our corresponding value is 45% (Table 1).

The site-specific rate constants obtained in this work (Table 1) also agree closely with the recommended values reported in Chemical Kinetics and Photochemical Data for Use in Atmospheric Studies, Evaluation No. 15 [22]. Our k_H,1_ is approximately 2% higher and k_H,2_ about 7% lower than the recommended values. The recommended total rate constant, 1.40 × 10^−10^ cm^3^ molecule^−1^ s^−1^, is only 1.5% lower than that measured here from substrate decay and roughly 3% higher than the sum of k_H,1_ and k_H,2_ at 298 K.

Furthermore, the most recent Chemical Kinetics and Photochemical Data for Use in Atmospheric Studies, Evaluation No. 19 [23] recommends the same total and site-specific rate constants for the C_3_H_8_ + Cl reaction as those reported in Evaluation No. 15.

The only study we found concerning the reaction of C_3_D_8_ with Cl atoms is that by Hitsuda et al. [18]. The values reported by Hitsuda et al. are in very good agreement with our corresponding data (Table 1 and Table 2). The difference in the total rate constant is only about 1%, while the deviation in the KIE (298 K) value does not exceed 8%.

The excellent agreement between our experimental data and previously reported kinetic parameters for the reactions of Cl atoms with C_3_H_8_ and C_3_D_8_ clearly demonstrates the reliability and accuracy of the present measurements. This consistency provides strong evidence that the experimental procedures were carefully executed and that the results obtained are robust. Consequently, we conclude that the rate constants for D-atom abstraction from C_3_D_8_, determined here for the first time over the temperature range 298–387 K, are reliable and experimentally well-founded.

From the perspective of theoretical chemistry, the overall process of hydrogen atom abstraction from the propane molecule (1) consists of three parallel reactions:

(27) $${CH}_{3}{CH}_{2}{CH}_{3}+Cl\overset{k_{RH1}}{\to }{CH}_{3}{CH}_{2}{CH}_{2}+HCl\;(\mathrm{lateral}\;\mathrm{H-atom})$$

(28) $${CH}_{3}{CH}_{2}{CH}_{3}+Cl\overset{k_{R{H1}^{\prime }}}{\to }{CH}_{3}{CH}_{2}{CH}_{2}+HCl\;(\mathrm{middle}\;\mathrm{H-atom})$$

(20) $${CH}_{3}{CH}_{2}{CH}_{3}+Cl\overset{k_{RH2}}{\to }{CH}_{3}CH{CH}_{3}+HCl$$

and for the deuterated system:

(29) $${CD}_{3}{CD}_{2}{CD}_{3}+Cl\overset{k_{RD1}}{\to }{CD}_{3}{CD}_{2}{CD}_{2}+DCl\;(\mathrm{lateral}\;\mathrm{D-atom})$$

(30) $${CD}_{3}{CD}_{2}{CD}_{3}+Cl\overset{k_{{RD1}^{\prime }}}{\to }{CD}_{3}{CD}_{2}{CD}_{2}+DCl\;(\mathrm{middle}\;\mathrm{D-atom})$$

(22) $${CD}_{3}{CD}_{2}{CD}_{3}+Cl\overset{k_{RD2}}{\to }{CD}_{3}CD{CD}_{3}+DCl$$

In this notation, transformations (27) and (29), as well as (28) and (30), correspond to the abstraction of the lateral and central H/D atoms, respectively, from the carbon atom of the –CH_3_ group. Conversely, the transformations involving the abstraction of the H/D atom from the –CH_2_– group of propane remained unchanged, and therefore the same numbering as in the experimental section of this paper is used.

**Figure 5:**
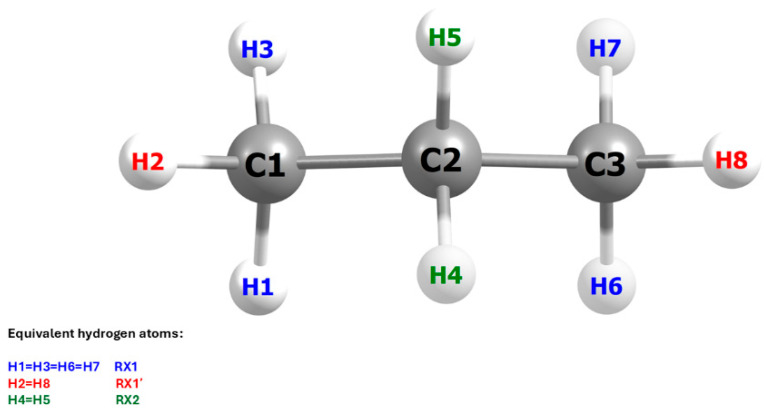
Schematic presentation of the propane molecule with the equivalent hydrogen atoms marked. Due to the molecular symmetry, only three out of eight individual reactions need to be considered to understand the mechanism of the investigated process.

This situation arises directly from the geometric structure of the propane molecule and its symmetry with respect to the carbon backbone plane. As illustrated in Figure 5, three elementary reactions must be considered in the computational analysis of the system, rather than two, as might be inferred from experimental studies. These studies, due to the free rotation of the methyl group, assume the equivalence of all hydrogen atoms within the group, without accounting for their spatial orientation relative to the plane in the C1–C2–C3 bond axis at the precise moment of the Cl radical attack. To reflect these differences, the notation used in Figure 5 distinguishes between the individual reactions: RX1 denotes the abstraction of the lateral hydrogen atom from the methyl group, RX1′ refers to the central hydrogen atom of the same group, and RX2 corresponds to the abstraction from the –CH_2_– group. The symbol X represents either a protium (H) or a deuterium (D) atom. This notation will be consistently applied throughout the present work.

**Figure 6:**
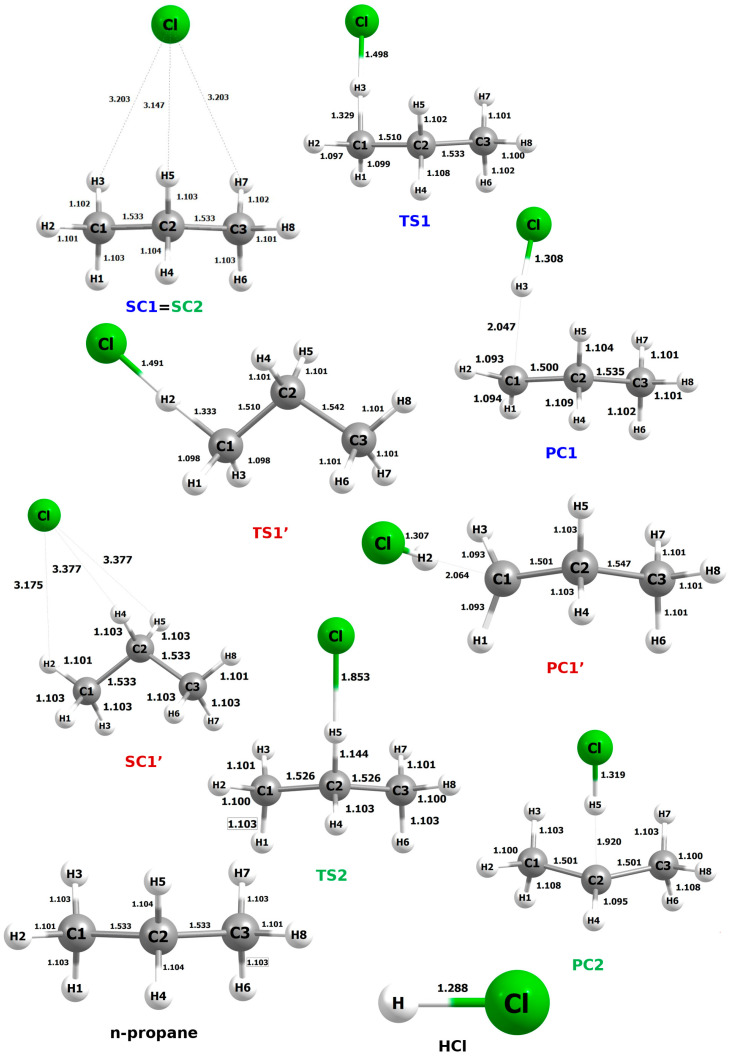
Optimized geometrical structures of the pre-reactive complexes (SCs), transition states (TSs), and post-reactive complexes. For comparison, the structures of the propane molecule and the hydrogen chloride molecule are also included. The systems include bond lengths and interaction distances.

The fully optimized geometrical structures of all systems for this reaction are shown in Figure 6. The SC, PC, and TS designations refer to the pre- and post-reactive complexes and the transition state, respectively.

The prereactive complex for reactions RX1 and RX2 corresponds to the same structure, denoted as SC1 = SC2. This results from very slight differences in the distances between atoms H3 and H5 and the attacking chlorine atom, which leads to stabilization of the system. This effect is further enhanced by the presence of an H7···Cl interaction, which can be considered equivalent to the H3···Cl interaction. The resulting highly symmetrical arrangement, stabilized by a triple non-covalent interaction, exhibits, as expected, the lowest possible energy.

In the PC2 structure, the H―Cl bond length is increased (ca. 1.30 Å) compared to that in an isolated HCl molecule (1.288 Å). This elongation is attributed to the persisting interaction between the hydrogen atom and the remaining propyl radical.

A shortening of the carbon–carbon bond is also observed as a result of the reaction. For processes RX1 and RX1′, this effect concerns the C1―C2 distance, whereas in RX2, due to the symmetry of the TS2 and PC2 structures, both C―C bonds shorten by an equal amount. These changes, however, are minimal and fall within the range of a few hundredths of an angstrom.

Among the analyzed transition state structures, TS2 stands out as the most distinctive. Compared to TS1 and TS1′, it exhibits the shortest C···H distance (1.144 Å), which is shorter by 0.185 Å and 0.189 Å, respectively. In contrast, the H···Cl distance in TS2 reaches 1.853 Å, representing an elongation of 0.355 Å and 0.362 Å relative to TS1 and TS1′, respectively. It is highly probable that TS2 displays reactivity features different from those of the other transition states, which will be discussed in detail in a subsequent section of the manuscript. Table S2 in the Supplementary Materials accompanying this manuscript presents, in addition to the fundamental geometrical parameters, the values of the imaginary frequencies, which confirm the correctness of the identified transition state structures.

A quantity that describes the character of a transition state, based solely on appropriately selected bond lengths (distances), is known as the *L-parameter*. It was defined in reference [24] as the following expression:

(31) $$L=\frac{\delta r\left(C-H\left(D\right)\right)}{\delta r\left(H\left(D\right)-Cl\right)}=\frac{r_{TS}\left(C-H\left(D\right)\right)-r_{react}\left(C-H\left(D\right)\right)}{r_{TS}\left(H\left(D\right)-Cl\right)-r_{react}\left(H\left(D\right)-Cl\right)}$$

The numerator refers to the relative elongation of the C―H (D) bond in the transition state (TS) with respect to its typical length in the reactant molecule prior to the onset of the reaction. The denominator, in turn, reflects the change in the length of the H(D)―Cl bond in the TS structure compared to its length in the free H(D)Cl molecule. The *L-parameter* is a dimensionless quantity characterizing the nature of the TS. If its value is less than 1, the transition state is considered reactant-like; for values greater than 1, it is regarded as product-like. According to the Hammond postulate [25], transition states in exothermic reactions tend to resemble the reactants, whereas in endothermic reactions, they are structurally closer to the products.

The values of the *L-parameter* for the transition states of the investigated elementary reactions are summarized in Table 4. Substituting the TS2 bond lengths into expression (31) yields

$$L_{TS2}=\frac{\left(1.144\;Å-1.104\;Å\right)}{\left(1.853\;Å-1.288\;Å\right)}\approx 0.07.$$

This low value indicates a reactant-like character for TS2, which, according to the Hammond postulate, suggests that the associated process (RX2) is more exergonic. All relationships concerning the geometrical parameters remain identical for the deuterated and non-deuterated systems.

**Figure 7:**
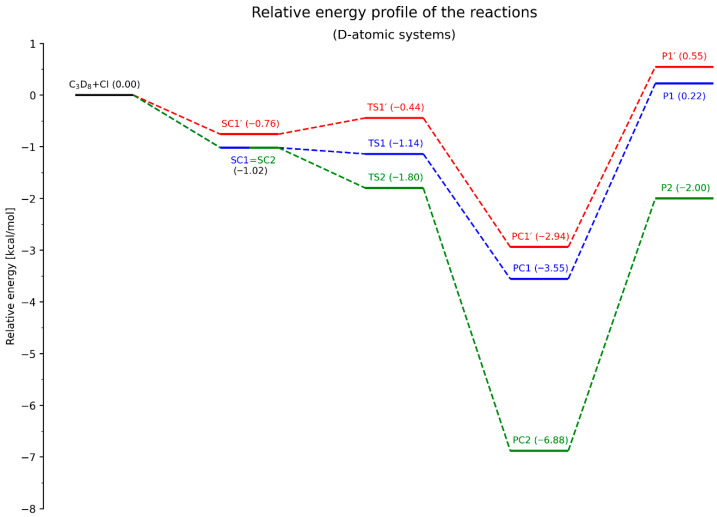
Energy diagram for the C_3_D_8_ + Cl reaction, illustrating the individual processes of D-atom abstraction. The relative energy values are given in kcal/mol.

**Figure 8:**
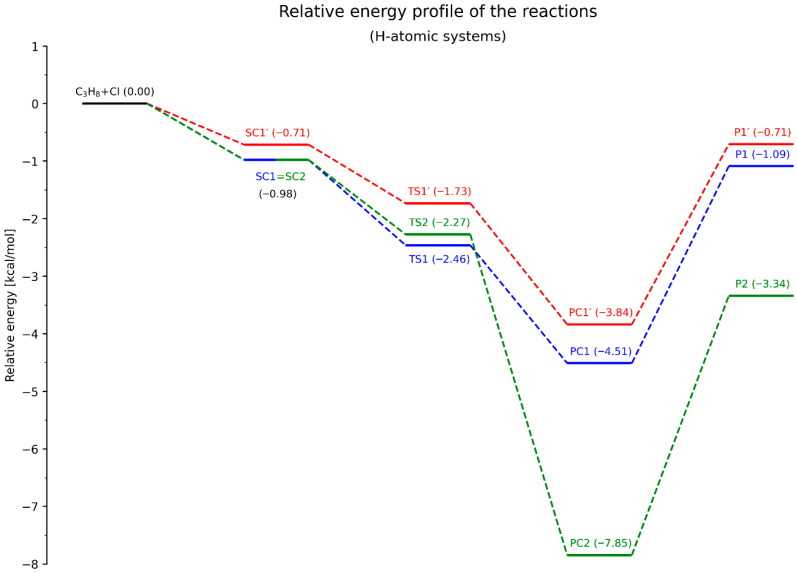
Energy diagram for the C_3_H_8_ + Cl reaction, illustrating the individual processes of H-atom abstraction. The relative energy values are given in kcal/mol.

The next step involves analyzing the energy profiles of all three elementary reactions. The energy diagrams for the systems containing deuterium and protium atoms are presented in Figure 7 and Figure 8, respectively.

The overall energetic profiles of all components of the studied reactions are generally similar for both isotopic forms. The same (vertical) ordering of the energy levels of SCs, PCs, and Ps is observed (where Ps denote the products, i.e., the propyl radical and hydrogen chloride molecule). However, differences arise in the case of the transition states.

For the deuterated system, the energetic ordering is TS1′ > TS1 > TS2, whereas in the non-deuterated system, TS2 is located between TS1′ and TS1. Moreover, the energy differences between all transition states (TSs) are significantly smaller in the non-deuterated system compared to the D-atomic system. This is particularly evident for TS1 and TS2, where ΔE_(TS2–TS1)_ amounts to only 0.19 kcal/mol, while in Figure 7 it reaches 0.66 kcal/mol. Additionally, the distribution of TS energy levels is more uniform in the deuterated system.

Let us consider the activation barriers and their associated implications. In the classical approach, the difference between the energy of the transition state and the sum of the energies of the reactants is defined as the activation energy barrier of the reaction. In this context, all examined processes (RX*N*, *N* = 1, 1′, 2) are barrierless, since the energy levels of the transition states (TSs) lie below the total energy of the reactants.

However, the situation changes in the deuterated system when analyzing the energy diagrams by considering the SCs as the initial states for the actual H/D-atom abstraction step and their corresponding transition structures. For the RD1′ transformation, the activation barrier is small but positive, amounting to 0.32 kcal/mol. In all other cases, regardless of the isotopic substitution, the individual reaction steps remain barrierless.

The absence of an activation barrier has significant consequences that are reflected in the kinetics of the chemical process. In such cases, an inverse temperature dependence of the reaction rate constants is typically observed [26,27].

Establishing a clear correlation between the values of the *L-parameter* and the energy diagrams representing the course of the studied processes is not a straightforward task. It should be noted that the *L-parameter* is based solely on structural features, in essence, only on differences in bond lengths (or interatomic interaction distances). There is no doubt that the energies of the individual stationary states, which correspond to specific points on the potential energy surface (PES), provide a much deeper and more comprehensive insight into the nature of the processes under investigation.

Among all the considered reaction pathways, only RX2 was classified, based on *L-parameter*, as an exergonic process, due to the reactant-like character of its transition state. Despite the absence of classical activation barriers, it can be stated that the largest energy difference occurs between PC2 (or P2, depending on the convention adopted) and SC2 (or the total energy of the reactants), which indeed makes RX2 the most exergonic of the processes analyzed.

Considering the energy levels of the final products (Ps), especially as shown in Figure 7 (where the values are positive), both RX1 and RX1′ appear to follow a significantly more endergonic course. In the case of energy differences between the SC and corresponding PC states within the same transformation, predicting the energetic character becomes less straightforward. Nonetheless, the energy differences between PC1 and PC1′ and SC1 = SC2 are clearly smaller than those between PC2 and SC1 = SC2, which further supports the lower exergonic character of RX1 and RX1′ compared to RX2.

Thus, although the correlation between *L-parameter* and the energetic profile is not direct, a consistent reflection of the structural relationships captured by the *L-parameter* can nevertheless be observed in the energetic characteristics of the individual reaction pathways involved in the interaction of the propane molecule with atomic chlorine.

It should be noted that the presented energy diagrams correspond to an absolute zero temperature. The described energy levels of each isotopic form refer to the vibrational zero-point energy (VZPE), and the energy differences between PCs and SCs (or between the sum of products and reactants) represent the reaction enthalpies (Δ_r_H°) at T = 0 K. The barrier heights, ΔE_TS-SC_ (ΔE_TS-reactants_), correspond to activation enthalpies (Δ^‡^H°(0 K)

To assess the impact of temperature on the mechanism, it is essential to consider thermodynamic functions, specifically the reaction enthalpy (Δ_r_H°) and Gibbs free energy (Δ_r_G°).

Table 5 summarizes the thermodynamic functions for all parallel channels at six temperatures (298.15–550 K). In accordance with the kinetic model used in this work, the reported reaction enthalpies and Gibbs free energies are defined for the microscopic step SC→PC (entrance complex to product complex). Both pre-reactive (SC) and post-reactive (PC) complexes are explicitly included in these definitions.

All analyzed values of Δ_r_H° and Δ_r_G° are negative. For the reaction enthalpy, this indicates an exergonic nature of the processes investigated. As the temperature increases, the value of Δ_r_H° systematically decreases across all transformations. In the deuterated system, this function assumes higher values at a given temperature than in the corresponding protiated ones. The order of decreasing exergonicity of the individual processes is as follows: RX2 > RX1 > RX1′ at all analyzed temperatures.

Although all reactions exhibit an exergonic character, the observed sequence is fully consistent, in qualitative terms, with the trends indicated by the *L-parameter* and the corresponding energy diagrams.

Gibbs free energy provides insight not only into the spontaneity but also the feasibility of a chemical reaction. The negative values of Δ_r_G° compiled in Table 5 indicate that each individual process proceeds spontaneously, and therefore so does the overall abstraction reaction.

As the temperature increases, the value of Δ_r_G° becomes less negative, which is fully expected, as the activation barrier simultaneously decreases. Unlike enthalpy, Gibbs free energy is lower in the protiated systems compared to their deuterated counterparts at the same temperature for a given process.

Based on the Δ_r_G° values, the most favorable reaction pathway proceeds via TS2. The next most feasible channels are the transformations RX1 and RX1′. This sequence is fully consistent with the previously discussed geometric and energetic characteristics. Undoubtedly, the formation of pre-reaction complexes is a key factor influencing the rate of the chemical reactions investigated. Expression (35), used to determine the rate constant, accounts for the contribution of these structures as stable starting complexes in the hydrogen atom abstraction step of each individual process.

For clarity, the Eyring–TST values reported here pertain to the SC→PC unimolecular step (first order) and are used for mechanistic diagnosis; second-order units and quantitative comparisons are reserved for experimental kinetics or for future treatments that include K_assoc_(T)/VTST.

For the gas-phase reaction between Cl atoms and propane (Cl + C_3_H_8_), long-range induction/dispersion interactions define a variational outer bottleneck; therefore, Eyring–TST referenced to the entrance complex (SC) provides an upper bound with respect to capture-limited kinetics unless that outer TS is included.

Table 6 reports first-order rate constants (10^11^ × *k* (SC→PC); units remain s^−1^) for all parallel channels and for the overall H/D-abstraction at six specified temperatures. These values are intended for qualitative use only (mechanism, channel ordering, temperature trends).

As expected, the rate constants (*k*) in the deuterated system are generally lower than in the non-deuterated one, and their variation with temperature is significantly reduced. For a given temperature, the highest values of k are observed for process RX2, followed by RX1, with RX1′ exhibiting the lowest rates. This trend is fully consistent with the reaction feasibility order, the degree of exoergicity, and the behavior of the *L-parameter*, which accounts for structural changes during the reaction pathway.

Another feature reflected in *k* is the disappearance of the activation barrier, previously discussed in the context of the energy diagrams of the studied process. For RD1′ (measured relative to the SC1′ complex), a positive activation energy was obtained (Figure 7), which results in a proportional dependence of *k*_RD1′_ on temperature.

Although for RD1 the corresponding difference is −0.12 kcal/mol, this value appears too small to reverse the observed temperature dependence of *k*_RD1_. In all other cases, the rate clearly decreases with increasing temperature. This trend is highlighted in red in Table 6 and also applies to the overall rate constant of the reaction. It may indicate that process RX2 contributes most significantly to the total abstraction process.

This can be verified using the concept of the *branching ratio* (*BR*). Following Cleaves et al. [28], the branching ratio (BR) is taken as the ratio of the rate constant for a given product channel to the total rate over all possible products. This operational definition is fully consistent with the IUPAC usage of “branching ratio” as the fractional contribution of an alternative product channel to the overall outcome [29]. The following formula can express the mathematical definition of BR for every individual reaction investigated:

(32) $$BR=\frac{RXN}{\sum_{i\in \left\{1,1^{\prime },2\right\}}^{3}\omega_{i}^{\left(N\right)}RX_{i}}$$

where *X* is the proper isotopic form, and *N* is the number of parallel reactions (*N* = 1, 1′, 2). $\omega_{i}^{\left(N\right)}$ is a weighting factor assigned to the reaction $RX_{i}$ in the denominator of the general expression for the fractional contribution of reaction RXN. $\omega_{i}^{\left(N\right)}$ is changeable and depends on *N* and *i*. Hence, this parameter can take on the values:

(33) $$\omega_{i}^{\left(N\right)}=\left\{\begin{array}{c} 1,if\;N=1\;and\;i=1\\ \begin{array}{c} \frac{1}{2},if\;N=1\;and\;i=1^{\prime }\;or\;i=2\\ \begin{array}{c} 2,if\;N=1^{\prime }\;or\;N=2\;and\;i=1\\ 1,if\;N=1^{\prime }\;or\;N=2\;and\;i=1^{\prime }\;or\;i=2 \end{array} \end{array} \end{array}\right.$$

**Figure 9:**
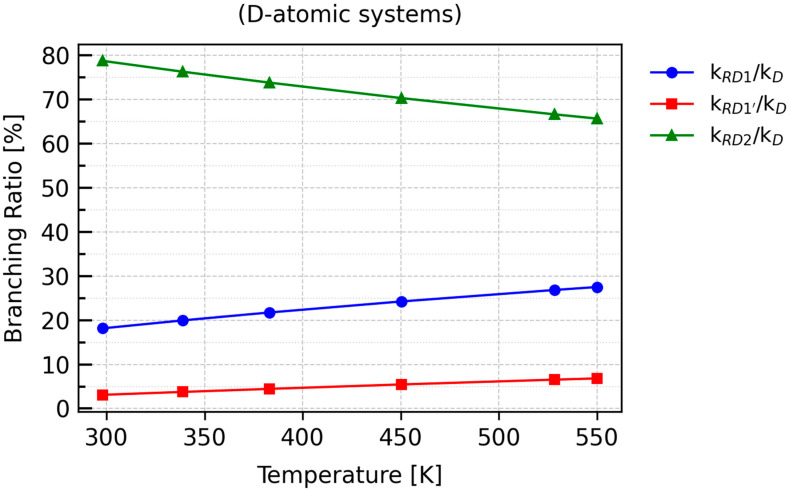
Plots of percentage branching ratio values as a function of temperature for individual reactions of D-atom abstraction from propane.

**Figure 10:**
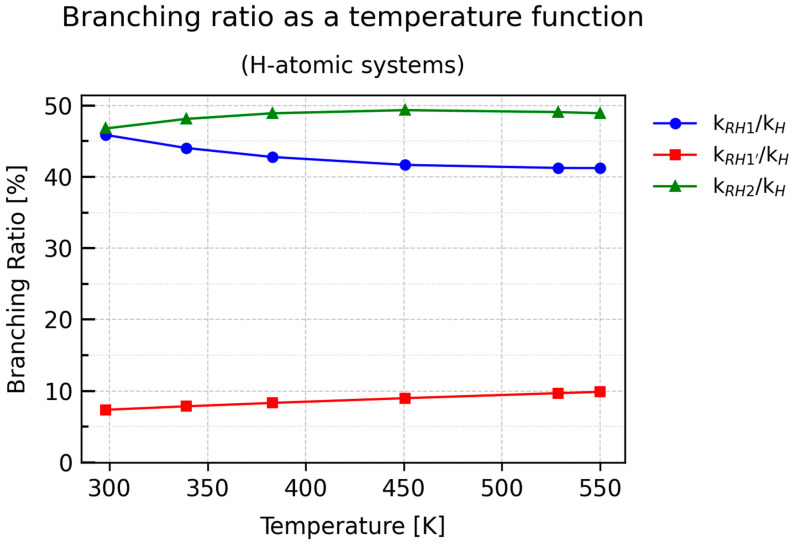
Plots of percentage branching ratio values as a function of temperature for individual reactions of H-atom abstraction from propane.

The percentage values of the *BR*s for all *RXN* reactions are presented in Table S3 of the Supplementary Materials. Their graphical representation is shown in Figure 9 and Figure 10, which correspond to the deuterated and protiated forms, respectively.

Both plots clearly indicate that RX2 is the most favored reaction channel. The next most probable pathway is RX1, while the contribution of RX1′ to the overall rate constant is less than 10%.

As the temperature increases, the contribution of *k*_RD2_ to the overall rate constant, *k*_D_, decreases to approximately 65%, which is consistent with the inverse temperature dependence for this transformation, as shown in Table 6. A similar trend is observed for RX1 and RX1′, whose *BR* plots exhibit positive slopes, indicating a proportional increase in their contributions with rising temperature.

Notably, the plots of *k*_RH2/_*k*_H_ and *k*_RH1/_*k*_H_ exhibit nonlinear behavior, in contrast to the nearly linear dependence of *k*_RH1′/_*k*_H_. At T = 298.15 K, both RH2 and RH1 contribute equally (approximately 46%), but their temperature-dependent trends evolve almost symmetrically: *k*_RH2/_*k*_H_ initially increases sharply and stabilizes around 49%, whereas *k*_RH1/_*k*_H_ decreases in a manner closely resembling the *BR* curve of RH2, reaching a plateau near 41%.

Thus, the plot of *k*_RH2/_*k*_H_ does not directly reflect the inverse temperature dependence expected from the rate constant of RH2. A similar pattern is seen for *k*_RH1/_*k*_H_. This can be attributed to two factors: first, the temperature dependence of the rate constants in the protiated system is significantly nonlinear; second, the overall rate constant *k*_H_ includes contributions from all parallel processes at a given temperature. Therefore, although the individual *k* values decrease with temperature, the ratio defining the branching ratio does not necessarily follow the same trend, as it represents the relative contribution of a given pathway to the total reaction rate rather than the absolute value of its rate constant.

The theoretical results referring to the reaction rate constants presented in this study show very good qualitative agreement with the previously reported experimental data. Although experimental measurements do not distinguish between individual reaction pathways within the methyl group, treating all hydrogen atoms as chemically equivalent, and cover only three temperatures (298 K, 334 K, and 387 K), a meaningful qualitative comparison is still possible.

The data presented in Table 1 and Table 2 clearly indicate an inverse temperature dependence of the rate constant, both for the individual reaction channels and for the overall process. This effect is particularly pronounced in the non-deuterated system and is consistent with the predictions of quantum chemical calculations, where the decrease in k is attributed to the absence of a classical activation barrier.

In the deuterated systems, the decline in k is less pronounced or tends to level off at higher temperatures (T = 334 K and 387 K), especially for reactions involving the methyl group. The agreement between these experimental observations and the mechanistic insight from theoretical modeling further supports the proposed interpretation of the temperature dependence.

In our gas-phase experiments, collisions are effectively isolated with respect to third-body effects. The residual discrepancy between experiment and theory primarily reflects the kinetic model: conventional Eyring–TST referenced to the entrance complex (SC) does not include the variational outer transition state that governs long-range capture and therefore yields upper-bound rate constants; further differences stem from the absence of variational/recrossing and tunneling corrections, anharmonic/hindered-rotor effects, and level-of-theory limitations.

## 3. Materials and Methods

### 3.1. Chemicals and Reagents

Helium (99.999%, Linde Gas, Biskupice Podgórne, Poland) was used as the carrier gas in chromatographic analyses.

Nitrogen (99.999%, Linde Gas, Biskupice Podgórne, Poland) served as an inert gas for the preparation of gas mixtures.

Chlorine (>99.5%, Aldrich, Pittsburg, CA, USA) was used as a photolytic source of chlorine.

Ethane (99.97%, Fluka Chemika, Buchs (St. Gallen), Switzerland) and propane (99.95%, PRAXAIR N.V., Antwerpen, Belgium) were used as reagent gases.

Propane-d_8_ (99% D, Isotec/Sigma-Aldrich, Miamisburg, OH, USA) was also used as a reagent gas.

Chloroethane (99.7%, PRAXAIR N.V., Oevel (Westerlo), Belgium), 1-chloropropane (99.5%, Fluka Chemika, Buchs (St. Gallen), Switzerland), and 2-chloropropane (>99%, Sigma-Aldrich, Sheboygan Falls, WI, USA) were used as calibration standards.

1-Chloropropane-d_7_ (99% D, CDN Isotopes, Ponte-Clare, QC, Canada) and 2-chloropropane-d_7_ (99.4%, CDN Isotopes, Ponte-Clare, QC, Canada) were used as deuterated calibration standards.

All reagents were of analytical or research grade and used without further purification.

### 3.2. Apparatus and Experimental Setup

A cylindrical quartz reactor (15 cm in length and 6 cm in diameter) was used for all experiments. The radiation source was a 100 W xenon (Xe) lamp. Its ultraviolet (UV) radiation, after passing through a monochromator and a slit, was directed into the reactor. The UV beam entered the reactor through its bottom base, traversed its entire length, exited through the top base, and was reflected back by a spherical mirror. After reflection, the beam re-entered the reactor at a small angle relative to the incident beam. The exact angle or point of beam entry was not critical, as the essential requirement was to maximize the optical path length of UV radiation within the reactor. The beam could also be introduced through a side wall, provided that it effectively passed through the reaction zone. If the optical path length was insufficient to achieve the desired degree of substrate conversion, the extent of photolysis could be adjusted by increasing the irradiation time or by broadening the incident beam.

From the entire UV spectrum emitted by the Xe lamp, radiation with a wavelength of approximately 330 nm was selected to irradiate the reaction mixture. The beam width was adjusted by varying the slit opening in the range of 5–20 nm. At a UV wavelength of 330 nm, the molar absorption coefficient for Cl_2_ reaches its maximum, resulting in the highest concentration of generated chlorine atoms [30].

### 3.3. Preparation of Gas Mixtures

Before the kinetic experiments, mixtures of C_3_H_8_ with C_2_H_6_ and C_3_D_8_ with C_2_H_6_ were prepared, each with an approximate reactant pressure ratio of 1:1, along with a 5% Cl_2_ mixture in N_2_. The prepared gas mixtures were stored in airtight, vacuum-sealed Pyrex flasks covered with black foil to prevent accidental exposure to light.

### 3.4. Experimental Procedure

The kinetic experiments were performed using the relative rate method based on substrate depletion. Each experiment consisted of a sequence of calibration–reaction–calibration runs. The calibration step was used to determine the initial substrate concentrations ([C_3_H_8_]_0_, [C_3_D_8_]_0_, or [C_2_H_6_]_0_), which were then used in calculations with Equations (6) and (7). Averaged concentrations obtained before and after the main experiment were applied.

Calibration involved filling the reactor with a mixture of C_3_H_8_ and C_2_H_6_ or C_3_D_8_ and C_2_H_6_ to a pressure of 4.00 Torr, followed by the addition of N_2_ to reach a total pressure of 100 Torr. The prepared mixture was analyzed chromatographically, and the peak areas for C_3_H_8_, C_3_D_8_, and C_2_H_6_ were directly proportional to their initial concentrations in the reactor.

In the main experiment, the reactor was filled with the same mixture (C_3_H_8_ and C_2_H_6_ or C_3_D_8_ and C_2_H_6_) to the same initial pressure as during calibration. A 5% Cl_2_/N_2_ mixture was then added to reach a total pressure of 100 Torr. After sealing the reactor, the mixture was irradiated with the Xe lamp radiation to photolyze Cl_2_ and generate chlorine atoms. The atomic chlorine reacted with propane (or deuterated propane) and ethane molecules.

The degree of substrate conversion was controlled by varying the photolysis duration (1–70 min) or adjusting the radiation beam width (±10 nm or ±20 nm).

In the kinetic studies of the reactions of hydrocarbons with chlorine, the effect of the reactor walls was negligible and did not influence the determined rate constants.

### 3.5. Theoretical Approach

The theoretical calculations were carried out on a broader temperature grid (298.15, 283, 339, 450, 528.5, 550 K) than the experimental set (298, 334, 387 K) to capture the temperature dependence of the mechanism more robustly and to reduce uncertainty in Eyring/Arrhenius fits. This range encompasses the experimental values and extends into a higher-temperature regime, allowing for the interrogation of entropic contributions, potential changes in the rate-determining step, and the smooth interpolation of activation parameters at the experimental points. All reactant, product and transition-state geometries (simple molecules and complexes) were fully optimised in the gas phase (simulated vacuum).

The nature of each stationary point on the potential energy surface (PES) was unequivocally confirmed through vibrational frequency analysis. Geometrical, energetic, and thermochemical parameters were obtained using the Gaussian 16 program package [31], employing Møller–Plesset second-order perturbation theory (MP2) [32,33,34,35,36,37] in conjunction with the aug-cc-pVDZ basis set [38]. This fully ab initio level captures dynamical electron correlation (including second-order dispersion) while avoiding functional-form dependence and thus provides a robust mechanistic baseline. Transition states were located via potential energy surface scans performed within the transition state search procedure [39]. To identify all possible reaction pathways and verify that the optimized stationary points indeed lie along the actual reaction coordinate, intrinsic reaction coordinate (IRC) calculations were carried out [40,41,42]. All computational results were graphically visualized using the Chemcraft program [43].

Theoretical reaction rate constants were determined from thermochemical data obtained via harmonic vibrational frequency analyses with Gaussian 16 [44]. The standard Gibbs free energy of activation, Δ^‡^G°, was evaluated for the ideal-gas standard state *p*° = 1 and inserted into the Eyring expression for a bimolecular gas-phase step:

To avoid ambiguity in the reaction order and units, conventional Eyring–TST is applied to the SC→TS→PC segment with the pre-reactive complex SC as the reference state. This microscopic step is unimolecular (SC→PC) and yields first-order rate constants (s^−1^). Conversion to observable second-order rate constants (cm^3^·molecule^−1^·s^−1^) requires the equilibrium association constant for forming SC, K_assoc_(T), such that *k*_bimol_(T) = K_assoc_(T) × *k*_uni_(SC→PC). In this study K_assoc_(T) is not fitted and VTST/capture is not employed; therefore, the reported *k*(SC→PC) values are used qualitatively (channel ranking, temperature trends) and are labeled pseudo-first-order.

Equation (34) shows the ideal bimolecular TST form:

(34) $$k\left(T,isotope\right)=\frac{k_{B}T}{hp{}^{\circ}}{\frac{RT}{p{}^{\circ}}e}^{-\frac{\Delta^{‡}G{}^{\circ}}{RT}}$$

In this work, Δ^‡^G° is referenced to SC, and the reported rate constants are first-order k (SC→PC):

(35) $$k_{SC\to PC}\left(T,isotope\right)=\frac{k_{B}T}{h}e^{-\frac{\Delta^{‡}G{}^{\circ}\left(SC\to TS\right)}{RT}}$$

All constants are used consistently; *R*, *k*_B_, and *h* are taken in a coherent unit system, and Δ^‡^G° values are converted accordingly.

For the systems studied, unlike typical Gaussian examples, the initial structures used in the rate-constant calculations were prereactive complexes (SC) rather than isolated radicals and atomic chlorine.

For barrierless channels (Δ^‡^G° ≤ 0), the reported *k*(SC→PC) values represent upper bounds. Mechanistic conclusions are drawn from relative ordering (e.g., RX2 > RX1 > RX1′) and temperature dependence rather than absolute magnitudes.

Referencing SC explicitly acknowledges an outer-region bottleneck governed by dispersion/induction interactions. Consequently, *k*(SC→PC) is not directly comparable to experimental second-order rate constants unless K_assoc_(T) or a VTST/capture treatment is introduced.

The explicit inclusion of both pre-reactive and post-reactive complexes is of critical importance, as these species constitute integral elements of the elementary reaction steps and are inseparably associated with the reaction mechanism. In particular, the presence of pre-reactive complexes has a substantial and determining impact on the overall reaction kinetics, faithfully representing the true physicochemical nature of the process.

## 4. Conclusions

Theoretical studies of the radical abstraction reaction of hydrogen and deuterium atoms from propane by atomic chlorine were aimed at gaining deeper insight into the reaction mechanism. The investigation was carried out in four main stages.

The first stage involved the optimization and analysis of the key geometrical structures participating in the process, including reactants, products, pre- and post-reaction complexes, and transition states. At this level, the use of the *L-parameter*, characterizing the nature of the transition state, indicated that RX2 is potentially the most exergonic of all considered reaction pathways.

In the second stage, potential energy diagrams were constructed for each individual reaction channel. These plots revealed that all parallel pathways, except for RD1′, proceed without a classical activation barrier. This suggests an inverse temperature dependence of the rate constant, which is typical for barrierless processes involving stabilized pre-reaction complexes.

Based on these energy profiles, the most probable sequence of reactivity was established. RX2 emerged as the most feasible pathway, followed by those proceeding through the TS1 and TS1′ transition states. This order was fully confirmed in the subsequent thermodynamic analysis, in which the values of reaction enthalpy (Δ_r_H°) and Gibbs free energy (Δ_r_G°) determined at six different temperatures indicated that all transformations are thermodynamically spontaneous, with RX2 being the most favorable and RX1′ the least.

The final stage of the study consisted of kinetic analysis, focused on the rate constants of both the individual steps and the overall abstraction process. As expected, all reactions in the non-deuterated system exhibited inverse temperature dependence. In contrast, in the deuterated systems, only RD1 and RD1′ showed an increase in k with rising temperature, particularly for RD1′; this trend may be attributed to a positive activation energy.

Absolute rate constants computed with quantum-chemical methods should be viewed as qualitative rather than strictly quantitative. Deviations from the experiment reflect not only methodological choices (level of theory, basis set, harmonic/rigid-rotor and conventional Eyring-TST approximations, omission of variational and tunneling corrections) but also the idealized isolated-system model, which neglects collisional energy transfer, pressure dependence, and environmental interactions.

It is explicitly stated that Eyring–TST here refers to the unimolecular SC→PC step (first order); second-order units are removed from *k*(SC→PC), and the values are labeled pseudo-first-order. Conversion to *k*_bimol_ requires K_assoc_(T) or a VTST/capture treatment.

The theoretical results showed excellent qualitative agreement with the experimental findings, ultimately confirming that hydrogen abstraction in the –CH_3_ group is less favorable and probable than in the –CH_2_– position. The established reactivity order serves as a final validation of the theoretical framework applied in this work, demonstrating the internal consistency of the outcomes across all stages of analysis—geometrical, energetic, thermodynamic, and kinetic. In the authors’ view, these findings contribute to a deeper understanding of the nature of the radical H/D-atom abstraction from propane initiated by atomic chlorine.

## Figures and Tables

**Table 1:** Experimentally determined relative rate constants for the hydrogen atom abstraction reaction, based on Equations (6), (19) and (23), along with the calculated absolute rate constants for these processes at 298, 334 and 387 K.

Temperature/K	k_H_/k_2_	k_H_ 10^−10^/ cm^3^ molecule^−1^ s^−1^	k_H,1_/k_2_	k_H,2_/k_2_	k_H,1_ 10^−11^/ cm^3^ molecule^−1^ s^−1^	k_H,2_ 10^−11^/ cm^3^ molecule^−1^ s^−1^	(k_H,1_ + k_H,2_) 10^−10^/ cm^3^ molecule^−1^ s^−1^
298	2.52 ± 0.05	$1.42_{-0.36}^{+0.38}$	1.09 ± 0.03	1.32 ± 0.04	$6.14_{-1.59}^{+1.69}$	$7.43_{-1.95}^{+2.01}$	$1.36_{-0.35}^{+0.37}$
334	2.30 ± 0.05	$1.34_{-0.44}^{+0.36}$	1.06 ± 0.06	1.23 ± 0.05	$6.19_{-1.80}^{+1.89}$	$7.18_{-1.98}^{+2.08}$	$1.34_{-0.38}^{+0.40}$
387	2.18 ± 0.03	$1.33_{-0.33}^{+0.35}$	0.98 ± 0.03	1.13 ± 0.05	$5.99_{-1.59}^{+1.68}$	$6.90_{-1.93}^{+2.05}$	$1.29_{-0.35}^{+0.37}$

Errors in relative reaction rates (columns 2, 4, 5) are 2σ experimental uncertainties. Errors in absolute rate constants (columns 3, 6, 7, 8) include experimental errors and uncertainties in the reference rate constant (2), calculated from Equation (26).

**Table 2:** Experimentally determined relative rate constants for the deuterium atom abstraction reaction, based on Equations (7), (24) and (25), along with the calculated absolute rate constants for these processes at 298, 334 and 387 K.

Temperature/K	k_D_/k_2_	k_D_ 10^11^/ cm^3^ molecule^−1^ s^−1^	k_D,1_/k_2_	k_D,2_/k_2_	k_D,1_ 10^11^/ cm^3^ molecule^−1^ s^−1^	k_D,2_ 10^11^/ cm^3^ molecule^−1^ s^−1^	(k_D,1_ + k_D,2_) 10^11^/ cm^3^ molecule^−1^ s^−1^
298	1.51 ± 0.02	$8.50_{-2.06}^{+2.25}$	0.46 ± 0.02	0.98 ± 0.03	$2.59_{-0.60}^{+0.64}$	$5.52_{-1.30}^{+1.36}$	$8.11_{-1.90}^{+2.00}$
334	1.45 ± 0.02	$8.46_{-2.08}^{+2.25}$	0.44 ± 0.06	0.93 ± 0.03	$2.57_{-0.60}^{+0.64}$	$5.43_{-1.28}^{+1.35}$	$8.00_{-1.88}^{+1.99}$
387	1.35 ± 0.02	$8.25_{-2.04}^{+2.21}$	0.44 ± 0.02	0.87 ± 0.03	$2.69_{-0.63}^{+0.67}$	$5.32_{-1.26}^{+1.33}$	$8.01_{-1.89}^{+2.00}$

Errors in relative reaction rates (columns 2, 4, 5) are 2σ experimental uncertainties. Errors in absolute rate constants (columns 3, 6, 7, 8) include experimental errors and uncertainties in the reference rate constant (2), calculated from Equation (26).

**Table 3:** The calculated values of the kinetic isotope effect (KIE), k_H_/k_D_, k_H,1_/k_D,1_ and k_H,2_/k_D,2_ were determined at temperatures of 298, 334, and 387 K.

Temperature/K	k_H_/k_2_	k_H,1_/k_2_	k_H,2_/k_2_	k_D_/k_2_	k_D,1_/k_2_	k_D,2_/k_2_	k_H_/k_D_	k_H,1_/k_D,1_	k_H,2_/k_D,2_
298	2.52 ± 0.05	1.09 ± 0.03	1.32 ± 0.04	1.51 ± 0.02	0.46 ± 0.02	0.98 ± 0.03	1.67 ± 0.06	2.37 ± 0.17	1.35 ± 0.08
334	2.30 ± 0.05	1.06 ± 0.06	1.23 ± 0.05	1.45 ± 0.02	0.44 ± 0.06	0.93 ± 0.03	1.59 ± 0.06	2.41 ± 0.46	1.32 ± 0.10
387	2.18 ± 0.03	0.98 ± 0.03	1.13 ± 0.05	1.35 ± 0.02	0.44 ± 0.02	0.87 ± 0.03	1.61 ± 0.05	2.23 ± 0.17	1.30 ± 0.10

The errors associated with the calculated KIE values (columns 8, 9, and 10) were estimated using the total differential method.

**Table 4:** The values of the *L-parameter* for the reaction of propane with atomic chlorine. The data refer to the individual processes studied of H/D-atom abstraction.

Transition State	*L-Parameter* Value
TS1	1.08
TS1′	1.14
TS2	0.07

**Table 5:** Reaction enthalpy and Gibbs free energy for the SC→PC step, Δ_r_H° (SC→PC) and Δ_r_G° (SC→PC), in kcal/mol, at the indicated temperatures (298.15–550 K). Values explicitly include the pre-reactive (SC) and post-reactive (PC) complexes. Channel labels refer here to the SC→PC step of the corresponding H/D-atom abstraction. Note that Equation (20) defines the bimolecular stoichiometry and is distinct from the SC→PC thermodynamic quantities reported in this table.

**Δ_r_H° [kcal/mol]**						
T [K]	RD1	RD1′	RD2	RH1	RH1′	RH2
298.15	−2.04	−1.64	−5.30	−3.20	−2.73	−6.41
339.00	−1.92	−1.51	−5.19	−3.06	−2.59	−6.29
383.00	−1.79	−1.38	−5.08	−2.92	−2.44	−6.17
450.50	−1.60	−1.20	−4.92	−2.70	−2.22	−5.99
528.50	−1.42	−1.02	−4.77	−2.47	−1.99	−5.79
550.00	−1.38	−0.97	−4.73	−2.41	−1.93	−5.74
**Δ_r_G° [kcal/mol]**						
T [K]	RD1	RD1′	RD2	RH1	RH1′	RH2
298.15	−2.41	−2.19	−5.76	−3.26	−2.98	−6.62
339.00	−2.46	−2.27	−5.83	−3.28	−3.02	−6.65
383.00	−2.54	−2.38	−5.92	−3.32	−3.09	−6.71
450.50	−2.69	−2.57	−6.09	−3.40	−3.22	−6.82
528.50	−2.90	−2.83	−6.30	−3.54	−3.41	−6.98
550.00	−2.96	−2.90	−6.36	−3.59	−3.47	−7.03

**Table 6:** The calculated values of rate constants for the individual processes and the overall reaction of propane with Cl atoms. k_D_ and k_H_ represent the overall rate constants for D- and H-atomic systems, respectively. Values that decrease with increasing temperature are highlighted in red.

T	*k*_RD1_	*k*_RD1′_	*k*_RD2_	*k*_D_	*k*_RH1_	*k*_RH1′_	*k*_RH2_	*k*_H_
[K]	10^11^∙*k* [s^−1^]							
298.15	84.81	29.07	735.84	1869.07	926.95	297.52	1890.67	8084.20
339.00	86.17	32.55	658.98	1727.74	698.15	248.73	1525.32	6340.70
383.00	88.83	36.40	603.51	1635.12	558.50	217.29	1276.93	5222.45
450.50	94.50	42.56	548.14	1559.38	441.70	190.47	1046.07	4239.91
528.50	102.54	50.00	509.12	1528.39	374.05	175.66	890.11	3627.72
550.00	104.95	52.07	501.15	1526.25	361.90	173.35	858.72	3511.75

## Data Availability

The data presented in this study are available on request from the corresponding author.

## Supplementary Materials

The following supporting information can be downloaded at: https://www.mdpi.com/article/10.3390/molecules30224406/s1, Table S1: Cartesian coordinates for all optimised geometrical structures included every stationary point necessary to describe the course of the reaction. Calculations were carried out at the MP2/aug-cc-pVDZ level of theory; Table S2: Basic geometrical parameters for the three most crucial stationary points of the studied processes. In addition, the values of the imaginary frequency of the transition state in cm^−1^ are included; Table S3: Exact branching ratio percentage values.
