# Supplementary material for: Experimental and Theoretical Studies on the Kinetics and Mechanism of the C3H8/C3D8 + Cl Reaction
Source: Molecules. 2025 Nov 14;30(22):4406. doi: 10.3390/molecules30224406 (PMC12654972; doi:10.3390/molecules30224406)
Supplement: Supplementary file 1 [file molecules-30-04406-s001.zip › molecules-3948257-supplementary.pdf]

Experimental and Theoretical Studies on the Kinetics and Mechanism of the C_3H_8/C_3D_8 + Cl Reaction

Łukasz Fojcik [1], Grzegorz Mierzwa [1], Zdzisław Latajka [2] and Dariusz Stanisław Sarzyński [3,*]

1. Institute of Environmental Protection – National Research Institute, ul. Słowicza 32, 02-170 Warszawa
2. Faculty of Chemistry, University of Wrocław, ul. Joliot-Curie 14, 50-383 Wrocław
3. Department and Institute of Basic Chemical Sciences, Wroclaw Medical University, ul. Borowska 211a, 50-556 Wrocław

SUPPLEMENTARY MATERIAL

Table of Contents

Table S1. **P2**

Cartesian coordinates for all optimised geometrical structures included every stationary point necessary to describe the course of the reaction. Calculations were carried out at the MP2/aug-cc-pVDZ level of theory.

Table S2. **P7**

Basic geometrical parameters for the three most crucial stationary points of the studied processes. In addition, the values of the imaginary frequency of the transition state in cm^{-1} are included.

Table S3. **P7**

Exact branching ratio percentage values.

Table S1. Cartesian coordinates for all optimised geometrical structures included every stationary point necessary to describe the course of the reaction. Calculations were carried out at the MP2/aug-cc-pVDZ level of theory.

Structure	Atom	X	Y	Z
Propane, C3H8	6	-1.468076000	-0.001982000	-3.845970000
	6	-1.468076000	-0.451647000	-1.342466000
	6	-1.468076000	0.616056000	-2.442827000
	1	-0.577768000	-0.635316000	-3.994653000
	1	-2.358385000	-0.635316000	-3.994653000
	1	-0.583848000	1.265866000	-2.326111000
	1	-2.352305000	1.265866000	-2.326111000
	1	-1.468076000	0.770900000	-4.630677000
	1	-2.358385000	-1.097136000	-1.423478000
	1	-0.577768000	-1.097136000	-1.423478000
	1	-1.468076000	-0.000154000	-0.337844000
SC1=SC2	6	-1.600116000	0.873506000	-0.608143000
	6	-0.920739000	1.265374000	0.708813000
	6	-1.106485000	0.196945000	1.792053000
	1	-1.184424000	-0.072051000	-0.993487000
	1	-2.683981000	0.731127000	-0.463117000
	1	0.156164000	1.425753000	0.532535000
	1	-1.329375000	2.227056000	1.063925000
	1	-1.460280000	1.643566000	-1.383192000
	1	-2.176509000	0.035570000	2.004360000
	1	-0.678544000	-0.765352000	1.466137000
	1	-0.613443000	0.482855000	2.734611000
	17	1.833313000	-1.027028000	-0.503658000
SC1’	6	0.006693000	-1.682328000	0.270136000
	6	0.002944000	-0.550391000	1.303878000
	6	0.005845000	-1.081629000	2.741973000

	1	0.899417000	-2.318584000	0.389886000
	1	-0.880961000	-2.325442000	0.390940000
	1	0.882926000	0.096186000	1.145850000
	1	-0.882181000	0.089385000	1.146898000
	1	0.004571000	-1.289533000	-0.758376000
	1	-0.881880000	-1.708271000	2.929678000
	1	0.898595000	-1.701410000	2.928622000
	1	0.003123000	-0.262143000	3.477966000
	17	-0.007929000	1.755855000	-1.657555000
PC1	6	-1.321200000	0.786507000	-0.613219000
	6	-0.962740000	1.284836000	0.755962000
	6	-1.074334000	0.201039000	1.836950000
	1	0.374864000	-0.334771000	-0.854362000
	1	-2.048312000	-0.022778000	-0.725589000
	1	0.056930000	1.708609000	0.742652000
	1	-1.630964000	2.131908000	1.014357000
	1	-1.116351000	1.399407000	-1.494906000
	1	-2.097147000	-0.207493000	1.872982000
	1	-0.380284000	-0.629041000	1.633082000
	1	-0.835789000	0.608687000	2.830970000
	17	1.525480000	-0.957579000	-0.869443000
PC1’	6	0.005333000	-1.341657000	0.249829000
	6	0.002663000	-0.457343000	1.462433000
	6	0.006647000	-1.280865000	2.771451000
	1	0.938638000	-1.795653000	-0.093271000
	1	-0.924858000	-1.802846000	-0.092129000
	1	0.887475000	0.200475000	1.450382000
	1	-0.887217000	0.193622000	1.451471000
	1	-0.001528000	0.224535000	-1.094599000

	1	-0.882391000	-1.927931000	2.828631000
	1	0.900727000	-1.921045000	2.827536000
	1	0.004602000	-0.612258000	3.646598000
	17	-0.006173000	1.304872000	-1.830007000
PC2	6	-1.144602000	-0.881138000	-1.472559000
	6	-1.351905000	-0.384752000	-0.071458000
	6	-1.153605000	-1.324138000	1.082096000
	1	-0.198397000	-1.440802000	-1.558402000
	1	-1.958178000	-1.575199000	-1.764004000
	1	0.306189000	0.567159000	0.099450000
	1	-2.013906000	0.475526000	0.075389000
	1	-1.133625000	-0.059060000	-2.203484000
	1	-1.968334000	-2.074835000	1.117271000
	1	-0.207348000	-1.881154000	0.980966000
	1	-1.148601000	-0.796164000	2.047188000
	17	1.514961000	1.087065000	0.193792000
TS1	6	-1.700363000	0.121553000	-3.807331000
	6	-1.609866000	0.716643000	-2.422576000
	6	-1.693425000	-0.338289000	-1.313559000
	1	-0.611921000	-0.630754000	-3.928101000
	1	-2.481945000	-0.633462000	-3.968923000
	1	-0.678901000	1.300486000	-2.334334000
	1	-2.443078000	1.440198000	-2.319548000
	1	-1.559997000	0.802229000	-4.656731000
	1	-2.636707000	-0.903866000	-1.383090000
	1	-0.858287000	-1.050636000	-1.395186000
	1	-1.650777000	0.131968000	-0.319634000
	17	0.650739000	-1.432190000	-4.005973000
TS1’	6	-1.474528000	-0.205298000	-3.765571000

	6	-1.476425000	0.493838000	-2.427347000
	6	-1.471734000	-0.535742000	-1.278909000
	1	-0.553762000	-0.734502000	-4.044843000
	1	-2.391500000	-0.741630000	-4.043723000
	1	-0.591227000	1.144485000	-2.348150000
	1	-2.366546000	1.137601000	-2.347070000
	1	-1.478859000	0.770756000	-4.673894000
	1	-2.361386000	-1.183270000	-1.325992000
	1	-0.577145000	-1.176351000	-1.327076000
	1	-1.473135000	-0.021852000	-0.305159000
	17	-1.483865000	1.914954000	-5.629213000
TS2	6	-1.866341000	-0.254034000	-3.896612000
	6	-1.920201000	0.343290000	-2.493871000
	6	-1.875217000	-0.691467000	-1.373768000
	1	-0.963536000	-0.872606000	-4.021418000
	1	-2.746990000	-0.896434000	-4.066478000
	1	-1.012021000	1.028319000	-2.371901000
	1	-2.775593000	1.029828000	-2.377842000
	1	-1.858923000	0.528395000	-4.670243000
	1	-2.756244000	-1.352512000	-1.436102000
	1	-0.972553000	-1.317018000	-1.458326000
	1	-1.874010000	-0.215164000	-0.381855000
	17	0.712287000	1.696943000	-2.249900000
P1 (radical)	6	-1.642554000	0.117368000	-3.814690000
	6	-1.486636000	-0.450825000	-1.356658000
	6	-1.505985000	0.654758000	-2.421763000
	1	-2.155665000	-0.829784000	-3.993137000
	1	-0.589762000	1.266473000	-2.347442000
	1	-2.343393000	1.349126000	-2.203497000

	1	-1.389072000	0.729661000	-4.681276000
	1	-2.410003000	-1.050665000	-1.403219000
	1	-0.633404000	-1.129357000	-1.513499000
	1	-1.407862000	-0.027897000	-0.343128000
P1' (radical)	6	-1.468076000	-0.150286000	-3.767819000
	6	-1.468076000	-0.453091000	-1.253209000
	6	-1.468076000	0.541651000	-2.437323000
	1	-0.534372000	-0.491529000	-4.217403000
	1	-2.401780000	-0.491529000	-4.217403000
	1	-0.581085000	1.192088000	-2.351972000
	1	-2.355068000	1.192088000	-2.351972000
	1	-2.358875000	-1.099694000	-1.287836000
	1	-0.577278000	-1.099694000	-1.287836000
	1	-1.468076000	0.084634000	-0.291366000
P2 (radical)	6	-1.441688000	-0.012380000	-3.872777000
	6	-1.441688000	-0.470724000	-1.320953000
	6	-1.282010000	0.482500000	-2.466815000
	1	-0.605037000	-0.679237000	-4.166104000
	1	-2.367636000	-0.603360000	-3.982116000
	1	-0.749409000	1.421774000	-2.298108000
	1	-1.471992000	0.813998000	-4.598746000
	1	-2.367636000	-1.062814000	-1.424111000
	1	-0.605037000	-1.197976000	-1.278028000
	1	-1.471992000	0.051362000	-0.352781000

Table S2. Basic geometrical parameters for the three most crucial stationary points of the studied processes. In addition, the values of the imaginary frequency of the transition state in cm^{-1} are included.

Reaction type	Energetic state	R_{C-H} [Å]	R_{H-Cl} [Å]	<C-H-Cl [°]	freq (cm^{-1}) H (for TS only)	freq (cm^{-1}) D (for TS only)
	SC1	1.103	3.147	117.4	-	-
RX1	PC1	2.047	1.308	172.1	-	-
	TS1	1.329	1.498	176.8	-833.29	-631.24
	SC1’	1.101	3.175	127.4	-	-
RX1’	PC1’	2.064	1.307	173.6	-	-
	TS1’	1.333	1.491	176.9	-858.68	-650.18
	SC3	1.103	3.147	117.4	-	-
RX2	PC3	1.920	1.319	173.4	-	-
	TS3	1.144	1.853	164.1	-184.25	-157.59

Table S3. Exact branching ratio percentage values.

T	k_{RD1}/k_D	$k_{RD1'}/k_D$	k_{RD2}/k_D	k_{RH1}/k_H	$k_{RH1'}/k_H$	k_{RH2}/k_H
298.15	18.15	3.11	78.74	45.86	7.36	46.77
339.00	19.95	3.77	76.28	44.04	7.85	48.11
383.00	21.73	4.45	73.82	42.78	8.32	48.90
450.50	24.24	5.46	70.30	41.67	8.98	49.34
528.50	26.84	6.54	66.62	41.24	9.68	49.07
550.00	27.51	6.82	65.67	41.22	9.87	48.91
